# Henk’s Atlas of Surgical Anatomy

**Published:** 1885-03

**Authors:** 


HENK'S ATLAS OF SURGICAL ANATOMY. *A Series of Plates Illustrating the Application of Anatomy to Medicine and Surgery. Translated by* W. A. ROTHAKER, M. D., *Lecturer on Pathological Anatomy, Miami Medical College.* A. E. WILDE & Co., *Cincinnati.*

This work will answer a good purpose for reference in refreshing the memory as to the relations of the different parts of the body. In the main, the illustrations are accurate and reliable, but we miss the coloring which makes Maclise's similar publication unsurpassed by any like work which has come under our inspection. It has some of the faults which are unavoidable, perhaps, by the attempt of the artist to make the different anatomical points show too plainly. The proper arrangement of the formation of the brachial plexus of nerves is not shown in any plate in the book.

Plate I shows the uvula as projecting below and beyond the upper edge of the epeglottis—a condition of things which would be far from pleasant to the owner of any such relation and one quite impossible.

Plate XIV, page 2. A section purporting to give the relations of the common carotid artery and internal jugular vein. These vessels are too far away from each other. Their distance increased in proportion, to bring it to the natural size, would give one a very erroneous idea of their relation, and if followed would very likely lead to the accident of wounding the vein. In the body they hug each other quite closely.

Plate XVIII is misleading in showing the relations existing between the common carotid arteries and jugular veins on the two sides of the neck. The rule seems to have been forgotten that the left veins approach their corresponding arteries, while the right veins diverge from them.

The most serious fault to our liking is the fact, that the short descriptive texts of the plates are all printed together in the front part of the book instead of each text going with its corresponding plate as could, it seems, easily have been arranged, thus saving much trouble of reference.

Plate XLI and LXI are both defective in that they fail to show the commencement or any portion of the internal iliac artery. It is not possible to look into the pelvis from any point which will display the arteries as here given, or fail to show some portion of the internal iliac. There is an appreciable difference between a ligation of the primitive iliac and the external iliac—through this plate no distinction could be made.

